# Dysfunction of type 1 and type 2 immune cells: a lesson from exhausted-like ILC2s and their activation-induced cell death

**DOI:** 10.1093/intimm/dxae032

**Published:** 2024-05-24

**Authors:** Takashi Ebihara, Toshiki Yamada, Akane Fuchimukai, Shunsuke Takasuga, Tentaro Endo, Takechiyo Yamada, Megumi Tatematsu

**Affiliations:** Department of Medical Biology, Akita University Graduate School of Medicine, Akita 010-8543, Japan; Center for Integrated Control, Epidemiology and Molecular Pathophysiology of Infectious Diseases, Akita University, Akita 010-8543, Japan; Department of Otorhinolaryngology, Head and Neck Surgery, Akita University Graduate School of Medicine, Akita 010-8543, Japan; Department of Medical Biology, Akita University Graduate School of Medicine, Akita 010-8543, Japan; Department of Medical Biology, Akita University Graduate School of Medicine, Akita 010-8543, Japan; Department of Otorhinolaryngology, Head and Neck Surgery, Akita University Graduate School of Medicine, Akita 010-8543, Japan; Department of Otorhinolaryngology, Head and Neck Surgery, Akita University Graduate School of Medicine, Akita 010-8543, Japan; Department of Medical Biology, Akita University Graduate School of Medicine, Akita 010-8543, Japan

**Keywords:** chronic allergy, chronic helminth infection, chronic viral infection, exhaustion, tumor

## Abstract

The concept of immune cell exhaustion/dysfunction has developed mainly to understand impaired type 1 immune responses, especially by CD8 T-cells against tumors or virus-infected cells, and has been applied to other lymphocytes. Natural killer (NK) cells and CD4 T cells support the efficient activation of CD8 T cells but exhibit dysfunctional phenotypes in tumor microenvironments and in chronic viral infections. In contrast, the concept of type 2 immune cell exhaustion/dysfunction is poorly established. Group 2 innate lymphoid cells (ILC2s) and T-helper 2 (Th2) cells are the major lymphocyte subsets that initiate and expand type 2 immune responses for antiparasitic immunity or allergy. In mouse models of chronic parasitic worm infections, Th2 cells display impaired type 2 immune responses. Chronic airway allergy induces exhausted-like ILC2s that quickly fall into activation-induced cell death to suppress exaggerated inflammation. Thus, the modes of exhaustion/dysfunction are quite diverse and rely on the types of inflammation and the cells. In this review, we summarize current knowledge of lymphocyte exhaustion/dysfunction in the context of type 1 and type 2 immune responses and discuss ILC2-specific regulatory mechanisms during chronic allergy.

## Teaser

Comparing ‘exhausted-like’ ILC2s with other exhausted lymphocyte subsets

## Introduction

Innate lymphoid cells (ILCs) can dictate the direction of immune responses and contribute to host protection and/or pathogenesis in inflammatory diseases and infections (1). On the basis of the types of helper cytokines ILCs produce and their requirements for transcription factors, ILCs can be classified into three groups and five subsets: T-bet-dependent group 1 ILCs, including natural killer (NK) cells and ILC1s; GATA-3-dependent group 2 ILCs, comprising ILC2s; and RORγt-dependent group 3 ILCs, consisting of ILC3s and lymphoid tissue inducer (LTi) cells (2). NK cells are highly motile and exert cytotoxicity against tumor cells and virus-infected cells, while other ILC subsets are tissue-resident and responsible for innate helper functions by early production of helper cytokines. Group 1 ILCs secrete CD4 T-helper 1 (Th1) cytokines, such as IFN-γ and TNF-α. These cytokines elicit type 1 immune responses, which protect against tumors and intracellular microbes by inducing activation of NK cells and CD8 cytotoxic T lymphocytes (1, 3, 4). ILC2s are innate counterparts of Th2 cells, as ILC2s produce IL-4, IL-5, IL-9, IL-10, and IL-13 to initiate type 2 immune responses which protect against multicellular parasites and toxins but can also mediate allergy (1, 5–10). ILC3s are a major innate source of type 3 cytokines, namely IL-17 and IL-22, and they promote type 3 immune responses against extracellular bacteria and fungi, similar to Th17 cells and/or Th22 cells (1, 11). LTi cells are necessary for the formation of secondary lymphoid organs in the fetus and are recognized as a subset of group 3 ILCs because LTi cells are dependent on RORγt and secrete type 3 cytokines (1, 2, 12).

Overwhelmed inflammation causes exhaustion/dysfunction of lymphocytes to protect hosts. The exhaustion of CD8 cytotoxic T cells was originally identified in the mice chronically infected with lymphocytic choriomeningitis virus (LCMV) (13). The concept of CD8 T-cell exhaustion is utilized to unleash dysfunctional CD8 cytotoxic T-cells in tumors by checkpoint inhibitors (14, 15). Th1 cells and NK cells contribute to the efficient induction of CD8 cytotoxic T cells but can become dysfunctional in tumors and chronic infections (16, 17). Thus, exhaustion/dysfunction of such type 1 immune cells results in uncontrollable tumor growth and persistent viral infections.

Initial ILC2 activation and successive Th2 induction promote type 2 inflammation. Chronic parasitic worm infections in mice lead to Th2 cells with a diminished capacity to produce Th2 cytokines (18–20). However, the behavior and fate of activated ILC2s during chronic allergy are poorly understood. We have recently identified how exaggerated type 2 inflammation during chronic allergy induces dysfunctional exhausted-like ILC2s that are removed from hosts via activation-induced cell death (AICD) (21–23). The process of ILC2 dysfunction leading to cell death is quite unique and different from that of other dysfunctional lymphocytes. In this article, we review the current understanding of immune cell exhaustion/dysfunction in lymphocytes including CD8 T cells, CD4 T cells, NK cells, and ILC2s during chronic type 1 or type 2 inflammation and discuss the differences and similarities among the lymphocytes with a special emphasis on exhausted-like ILC2s and their AICD.

## CD8 T-cell exhaustion

During tumor development and chronic viral infections, CD8 T-cells differentiate into an exhausted or dysfunctional state, which is accompanied by increased expression of inhibitory “immune checkpoint” receptors such as PD-1, LAG-3, TIM-3, CTLA-4, and TIGIT on the cell surface (24). Continuous T-cell receptor (TCR) stimulation induces CD8 T-cell dysfunction by increased inhibitory cellular signaling from the inhibitory receptors, leading to an impaired ability to proliferate and produce type 1 inflammatory cytokines such as IFN-γ, IL-2, and TNF-α; however, the inhibitory functions of these receptors can be blocked by antibodies (immune checkpoint inhibition) that are used for immunotherapy (14, 15, 24).

**Figure 1.**
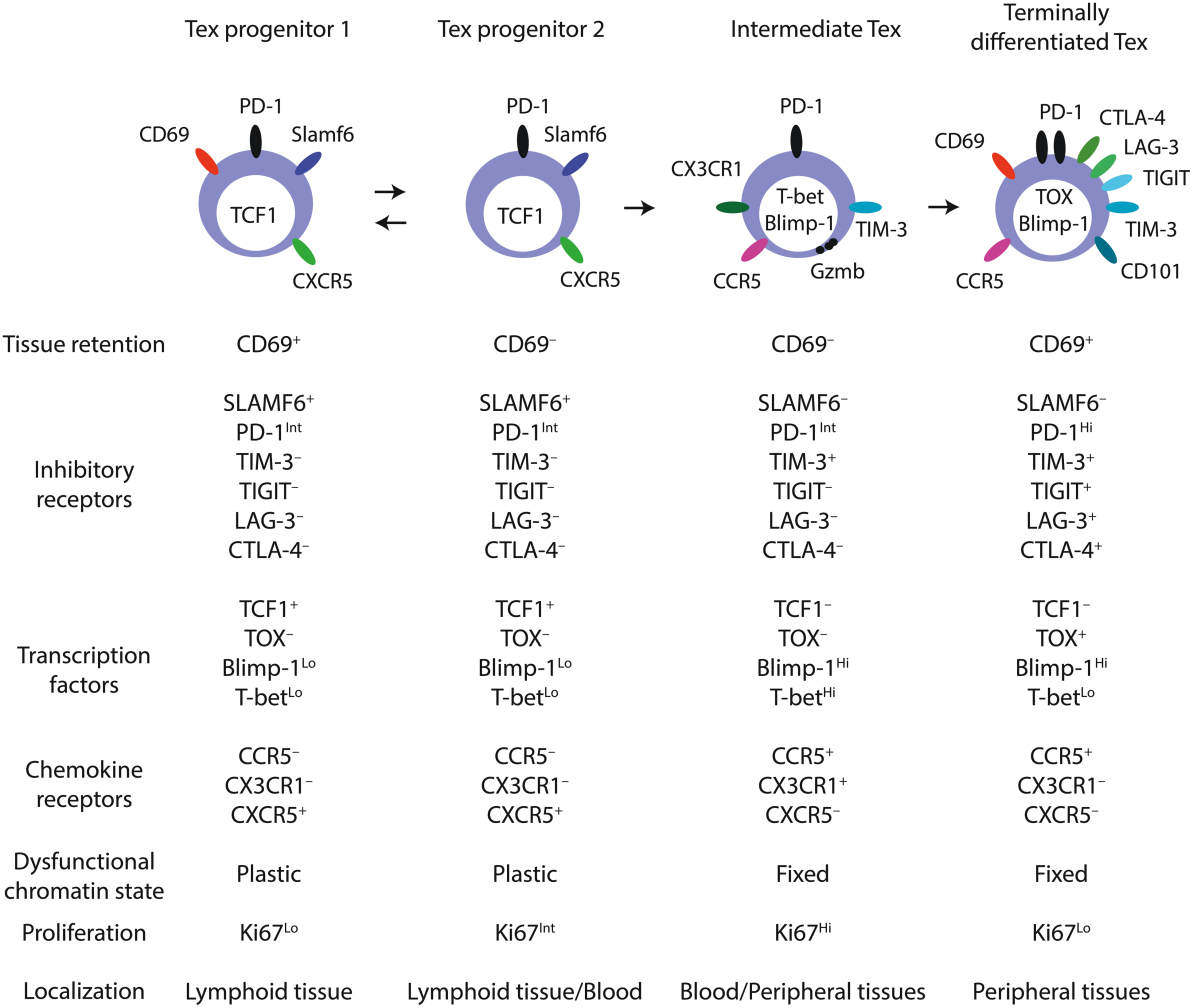
Differentiation of exhausted CD8 T cells. Exhausted CD8 T cells (Tex) are classified into four subsets based on expression of CD69, SLAMF6, and other markers. The expression of inhibitory checkpoint receptors increases concurrently with Tex differentiation. Tex progenitor 1 and progenitor 2 express TCF1, which inhibits Blimp-1 and TIM-3. Tex progenitor 1 is a subset of quiescent progenitors in lymphoid tissues. Tex progenitor 2 are proliferative and located in lymphoid tissues and the circulation. Intermediate Tex downregulates TCF1 and expresses Blimp-1 and T-bet, which confers effector function onto the cells by increasing granzyme B (Gzmb) and perforin expression. Terminally differentiated Tex are characterized by TOX expression, which highly induces exhaustion-related gene expression.

The differentiation process of dysfunctional CD8 T cells is precisely reviewed elsewhere (14, 15, 24). Briefly, four exhausted CD8 T-cell subsets have been identified, determined by CD69 and SLAMF6 expression (Fig. 1) (25). CD69^+^ SLAMF6^+^ exhausted T progenitor 1 cells and CD69^−^ SLAMF6^+^ exhausted T progenitor 2 cells can respond to PD-1 blockade therapy for rejuvenation (25, 26). Both progenitors express TCF1 to inhibit expression of TIM-3 and Blimp-1 but promote that of CXCR5 and Bcl6, which counteracts Blimp-1 (27–30). Increased Blimp-1 expression induces the loss of TCF1 and increases PD-1 and LAG-3 to drive further exhaustion to CD69^−^ SLAMF6^−^ intermediate and CD69^+^ SLAMF6^−^ terminally differentiated exhausted T cells (25). T-bet is highly upregulated in intermediate exhausted T cells and is implicated in the efficient proliferation of the cells. Intermediate exhausted T cells are boundary cells that retain the ability to regain effector function (25, 31). Terminally differentiated exhausted T-cells express TOX and accumulate in peripheral tissues, losing effector function progressively (25). TOX is required for the upregulation of inhibitory checkpoint receptors, decreased functions, and epigenetic modification for the dysfunction (32, 33). Thus, CD8 T-cell dysfunction is a stepwise process marked by differential expression of inhibitory receptors, chemokine receptors, and transcription factors.

The chromatin state within dysfunctional cells is associated with the differential expression of exhaustion-related genes (33–35). For example, in the gene loci of *Ifng* and *Pdcd1*, there are some open chromatin loci specific to exhausted CD8 T cells but not effector and memory T cells. These dysfunctional chromatin states are reversible in CD38^Lo^ CD101^Lo^ tumor-specific exhausted T cells but not CD38^Hi^ CD101^Hi^ exhausted T cells (36). CD38 is expressed in terminally exhausted T-cells and negatively relates to TCF1 expression (Fig. 1). Thus, the epigenetic state of exhausted CD8 T-cells governs the phenotype and their response to immune checkpoint inhibition.

## CD4 T-cell exhaustion during chronic type 1 inflammation

**Figure 2.**
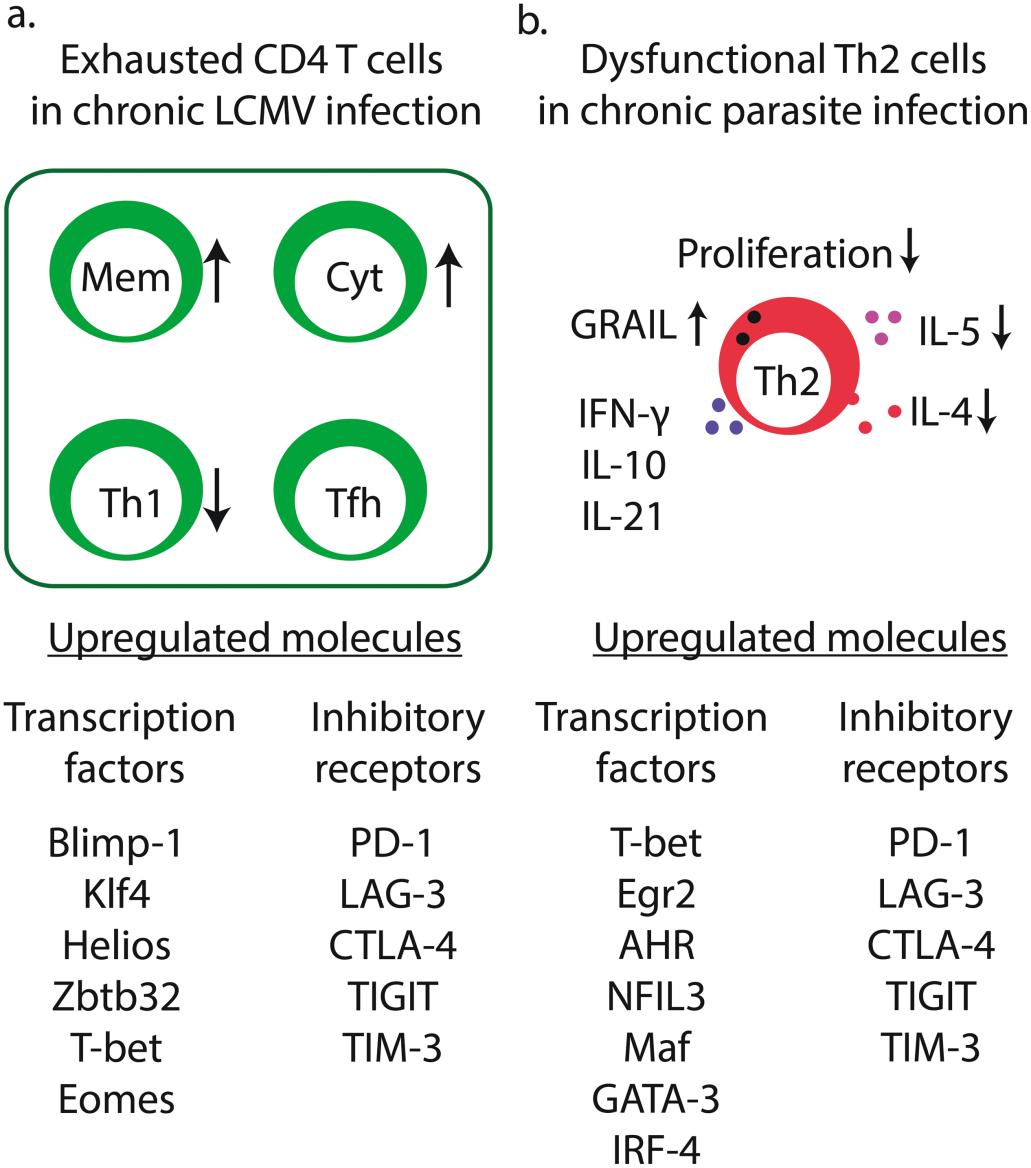
Differentiation of exhausted CD4 T cells. (a) In chronic LCMV infection, bulk CD4 T cells expressing inhibitory checkpoint receptors exhibit decreased production of the cytokines IFN-γ, IL-2, and TNF-α and increased levels of Blimp-1, Klf4, Helios, Zbtb32, T-bet, and Eomes compared to the CD4 T cells in acute LCMV infection. Single-cell analysis indicated that Th1 cell differentiation is hampered despite an increased frequency of memory (Mem) T cells and cytotoxic (Cyt) T cells. Tfh cells include pre-Tfh, Tfh1, and Tfh2 cells. The frequency of the precursor Tfh cells may be slightly increased among the dysfunctional CD4 T cells, despite no preferential Tfh1 and Tfh2 skewing. (b) In chronic parasite infection, exhausted/dysfunctional Th2 cells have been identified in IL-4-reporter mice. The chronic infection leads to decreased levels of IL-4 and IL-5 production and proliferation but induces T-bet and c-Maf expression in the dysfunctional Th2 cells, which have the potential to produce IFN-γ, IL-10, and IL-21.

CD4 T-cell exhaustion has been mainly studied using chronic LCMV infection in mice (Fig. 2a) (16, 17). However, the definition of CD4 T-cell exhaustion has not been well established. Consensus markers to identify exhausted Th1 cells are lacking. Chronic LCMV infection induces dysfunctional antigen-specific CD4 T cells that are characterized by high expression of inhibitory checkpoint receptors such as PD-1, LAG-3, and CTLA-4, and decreased production of the cytokines IFN-γ, IL-2, and TNF-α (16, 37).

The emergence of exhausted CD4 T cells is supported by mechanisms similar to CD8 T-cell exhaustion and by mechanisms specific to exhausted CD4 T cells (16, 38). Exhausted CD4 T cells upregulate Blimp-1, BATF, T-bet, and Eomes, which are also involved in CD8 T-cell exhaustion (16, 39). Upregulated transcription factors specific to exhausted CD4 T cells, not to exhausted CD8 T cells, are Helios and Klf4 (16). ThPOK represses CD4 T-cell exhaustion by suppressing the expression of Runx3 and Blimp-1, which drive gene expression for exhaustion (40). Thus, these transcription factors mediate CD4 T-cell dysfunction in bulk.

CD4 T-cell dysfunction may be partly due to the skewing toward another T-helper cell population—T follicular-helper (Tfh) cells—during chronic LCMV infection (41). Continuous TCR stimulation drives Tfh differentiation by increased expression of the Tfh-associated master regulator, Bcl6. Meanwhile, recent single-cell technology elucidated the transcriptional landscape and heterogeneity of exhausted CD4 T cells in chronic LCMV infection (42). Antigen-specific exhausted CD4 T cells are classified into eight subsets including Th1 cells, Tfh cells, memory T cells, and cytotoxic CD4 T cells. Attenuation of Th1 activity is a result of a decreased Th1 cell population and a global decrease in the Th1 gene profile among all CD4 subsets. Tfh signature genes are upregulated in the Th1 subsets while the frequency of the Tfh-related population including precursor Tfh (pre-Tfh) cells, Tfh1 cells, and Tfh2 cells is not increased in total. In contrast, memory CD4 T cells and cytotoxic CD4 T cells are increased in chronic LCMV infection. Therefore, CD4 T-cell dysfunction is attributed to integrated immune responses by heterogenous CD4 T-cell populations. The differentiation and epigenetic landscape of exhausted CD4 T-cells in chronic LCMV infection remains elusive.

## NK cell exhaustion

**Figure 3.**
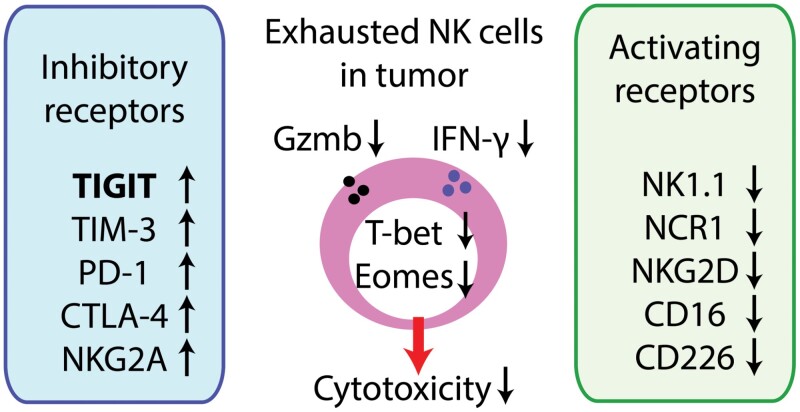
NK cell exhaustion in tumors. NK cell dysfunction is induced in tumors expressing any NK-activating ligands and in tumors that downregulate MHC class I expression. NK cell effector functions such as IFN-γ production and cytotoxicity via granzyme B (Gzmb) are impaired in the exhausted NK cells. Downregulated T-bet and Eomes in part mediate the functional disability, which is reversed by forced expression of Eomes. In general, NK cell activating receptors are downregulated in the exhausted NK cells. In contrast, several inhibitory receptors are increased and contribute to the dysfunction. TIGIT seems to be the most highly induced among inhibitory checkpoint receptors in the dysfunctional NK cells.

Many tumors or chronic infections lead to NK cell exhaustion/dysfunction characterized by impaired cytotoxicity and decreased IFN-γ production (Fig. 3) (43–49). NK cell activity is determined by integrated stimuli from inhibitory receptors such as NKG2A, TIGIT, the Ly49 family (receptors of self-MHC I in mice), and the KIR family (receptors of self-MHC I in humans) and activating receptors such as NKG2D, NCRs, CD16, and CD226 (43). In most cases, exhausted NK cells express decreased levels of activating receptors including NKG2D and NCRs, alongside increased levels of inhibitory checkpoint receptors (Fig. 3) (47). Tumor cells expressing ligands that activate NK cells or tumor cells without self-MHC class I induce NK cell proliferation accompanied by a rapid loss of NK cell function (50, 51). Exhausted NK cells exhibit decreased expression of Eomes and T-bet, both of which govern NK cell differentiation and function. Single-cell RNA-sequencing assays showed that exhausted NK cells are quite diverse in murine and human tumors (52). In murine tumors, dysfunctional NK cells are preferentially localized in the CD27^Hi^ CD11b^Lo^ immature population, which expresses decreased mRNA levels of *Klrb1c* (NK1.1), *Ncr1*, *Itga2* (CD49b), and *Ly49s* but increased mRNA levels of *Kit*, *Pdcd1*, *Tigit*, and *Ctla4*.

Some studies emphasized the role of PD-1 in NK cell exhaustion and its reversion (48, 49). However, a recent study by Judge and colleagues suggests that PD-1 expression in activated murine and human NK cells is minimal and that TIGIT is the most induced checkpoint molecule among co-inhibitory receptors including PD-1, TIM-3, and LAG-3 (53). CD155, the most potent TIGIT ligand, is expressed by many human and murine tumor cells and dampens NK cell activity. Therefore, TIGIT blockade alleviates NK cell exhaustion and limits tumor growth (54). However, TIGIT is not a definitive marker of NK cell exhaustion because murine steady-state NK cells express TIGIT, which is involved in NK cell education/licensing (55). Reversal of NK cell exhaustion is feasible and can be achieved by forced expression of Eomes, by blockade of inhibitory receptors, by cytokine stimulation with IL-12/IL-18 or a mutant form of IL-2, and by inhibition of the ATM DNA-damage-repair pathway (51, 56–58).

Torcellan *et al.* (59) have recently identified TCF1^+^ ILC1s derived from circulating NK cells in a model of acute viral infection in the skin. Circulating NK cells migrate to the skin upon viral infection and differentiate into either TCF1^+^ CD69^+^ ILC1s or Blimp-1^+^ NK cells. TCF1^+^ ILC1s reside in the skin after resolution and respond to a second challenge by the virus robustly. In contrast, Blimp1^+^ NK cells can return to the circulation. Interestingly, differentiation of the TCF1^+^ ILC1s does not depend on Hobit, which is necessary for tissue residency of ILC1s. TCF1 is required for memory responses by CD8 T-cells during the convalescent phase, as well as for differentiation of progenitor exhausted CD8 T-cells during chronic viral infection (25). Therefore, chronic viral infections in the skin may induce a dysfunctional state in TCF1^+^ ILC1s, endowing them with progenitor-like properties.

## CD4 T-cell exhaustion/dysfunction during chronic type 2 inflammation

In contrast to the exhaustion of type 1 immune cells (i.e. cells that mediate type 1 immune responses), knowledge of Th2 cell exhaustion is very limited. In the setting of chronic helminth infection, Th2 cells down-modulate their functional activity (Fig. 2b) (18–20). During chronic *Schistosoma mansoni* infection, IL-4-producing Th2 cells rapidly increase and reach a plateau level maintaining their proliferative ability at 8 weeks post-infection (18). However, at 16 weeks post-infection, Th2 cells exhibit decreased proliferation, suggesting that chronic *Schistosoma* infection induces Th2 exhaustion/dysfunction. The E3 ubiquitin ligase GRAIL is upregulated in the exhausted Th2 cells and is necessary for hyporesponsiveness. GRAIL may contribute to Th2 exhaustion by ubiquitinating Rho GDP-dissociation inhibitor family members, which inhibit proliferation (60).

Taylor and colleagues published two papers, using IL-4gfp 4get reporter mice, to clarify how dysfunctional Th2 cells attenuate chronic inflammation due to helminths (19, 20). They followed the GFP^+^ CD4 T-cells up to 60 days post-infection and found that the number of GFP^+^ CD4 T-cells was continuously increasing. However, the actual expression of IL-4 and IL-5 proteins returned to the levels in naïve Th2 cells at 40 days post-infection and further decreased at 60 days post-infection. The Th2 dysfunction is associated with high PD-1 expression and can be reversed by blockade of PD-1 or PD-L1 (a PD-1 ligand) (19). Transcriptional analysis suggested that the dysfunctional Th2 cells retain the Th2 gene expression signature but increase the type 1 immune potential by upregulating T-bet, which enables the cells to produce IFN-γ (Fig. 2b) (20). Global transcription profiles of the dysfunctional Th2 cells are similar to T-cell anergy because they express anergy markers including *Egr2* and *Grail*. The dysfunctional Th2 cells express Blimp-1, which may induce PD-1 in the cells as observed in exhausted CD4 T-cells in chronic LCMV infections. Upregulated genes in the dysfunctional Th2 cells also include tolerance-related genes such as *Lag3*, *Tigit*, *Havcr2*, *Icos*, *Il10*, *Il21*, *Maf*, *Nfil3*, and *Ahr*. Increased c-MAF expression may be responsible for IL-10 and IL-21 production by the dysfunctional Th2 cells (61) (Fig. 2b). Thus, dysfunctional Th2 cells have a distinct gene expression profile for their hyporesponsiveness.

## Identification of “exhausted-like” ILC2s

ILC2s receive numerous stimulatory and inhibitory signals from tissue-specific environments and are dynamically regulated by cytokines, lipid mediators, sex hormones, neuropeptides, and direct contact with other immune cells in the tissues (5, 62). The most potent activating signals seem to be made via IL-33, IL-25, and TSLP because the absence of IL-25/TSLPR/ST2(IL-33R) mostly abrogates ILC2 proliferation in mice infected with helminths or intranasally treated with house dust mite (63). Especially, IL-33 strongly stimulates ILC2s and allows the cells to proliferate for at least several months while maintaining their high ability to produce IL-5 and IL-13 *in vitro* (64).

ILC2 activity is also supported by direct cell–cell contact. For example, ILC2s express ICOS and ICOS ligand, both of which promote ILC2 proliferation and cytokine production by *trans*-interactions among ILC2s (65). Death receptor 3 is a cell surface receptor expressed on ILC2s and induces ILC2 activation through TL1A expressed in macrophages and dendritic cells activated by Toll-like receptor (TLR) agonists (66). ILC2s also express GITR, whose cellular signaling induces IL-9 via interaction with GITRL expressed in many activated lymphocytes, macrophages, and dendritic cells to support ILC2 activity (67, 68).

To suppress ILC2 proliferation and activity, inhibitory soluble factors against ILC2s are secreted. They include type 1/2 IFNs, IL-27, IL-10, adrenaline/noradrenaline, CGRP, testosterone, and lipid mediators such as PGE2 and PGI2 (5, 62, 69–74). For example, a lack of IFN-γ (i.e. type 2 IFN) in the RAG2-deficient background induces the development of spontaneous pulmonary fibrosis due to increased IL-13-producing ILC2s (75).

Activated ILC2s express inhibitory receptors such as KLRG1 and PD-1 to suppress ILC2 effector functions (76). Agonistic stimulation of PD-1 is a promising approach to treat chronic allergy because a human agonist of PD-1 ameliorates airway allergy in humanized mice (77). Nevertheless, KLRG1 and PD-1 are not markers for dysfunctional ILC2s. Expression of KLRG1 reflects the maturation status as well as activation in ILC2s because KLRG1 is not expressed in bone marrow ILC2 progenitors but is expressed in peripheral ILC2s (78). In contrast, PD-1 expression is barely detected in steady-state ILC2s without inflammation but is induced in KLRG1^+^ ILC2s after activation (21, 76). Whereas PD-1 on the activated ILC2s has somewhat inhibitory effects upon the pool of activated ILC2s (76), the number of lung PD-1^+^ ILC2s stably increases and never reaches a plateau phase during chronic airway allergy induced by nasal papain treatments for 3 weeks (21). Thus, ILC2s apparently do not undergo exhaustion due to overstimulation.

The concept of “exhausted-like” ILC2s comes from a study of Runx proteins in ILC2s (23). Runx proteins are a transcription-factor family comprising Runx1, Runx2, Runx3, and their binding partner Cbfβ (79, 80). Because heterodimer formation between Cbfβ with any of the other Runx proteins is required for DNA-binding, the absence of Cbfβ abolishes all Runx functions. Runx proteins are differentially expressed by ILC subsets and regulate ILC differentiation (23, 81). NK cells and ILC1s express a high level of Runx3, which contributes to the survival of the cells partly by upregulating the anti-apoptotic molecule Bcl2. Intermediate expression of Runx3 is detected in ILC3s and is indispensable for expression of the ILC3 master regulator RORγt. ILC2s express a high level of Runx1 instead of Runx3. When *Cbfb* is genetically deleted in ILC2s, the ILC2s normally populate tissues but exhibit an activated phenotype characterized by increased expression of IL-5 and KLRG1, a marker for maturation/activation of ILC2s (23). Because Runx proteins antagonize the ILC2 master regulator GATA-3 by direct binding, the absence of Runx proteins may increase free GATA-3 in the cells, leading to the activation of ILC2s. Consistent with this, many GATA-3-inducible genes are upregulated in the ILC2s lacking Cbfβ.

**Figure 4.**
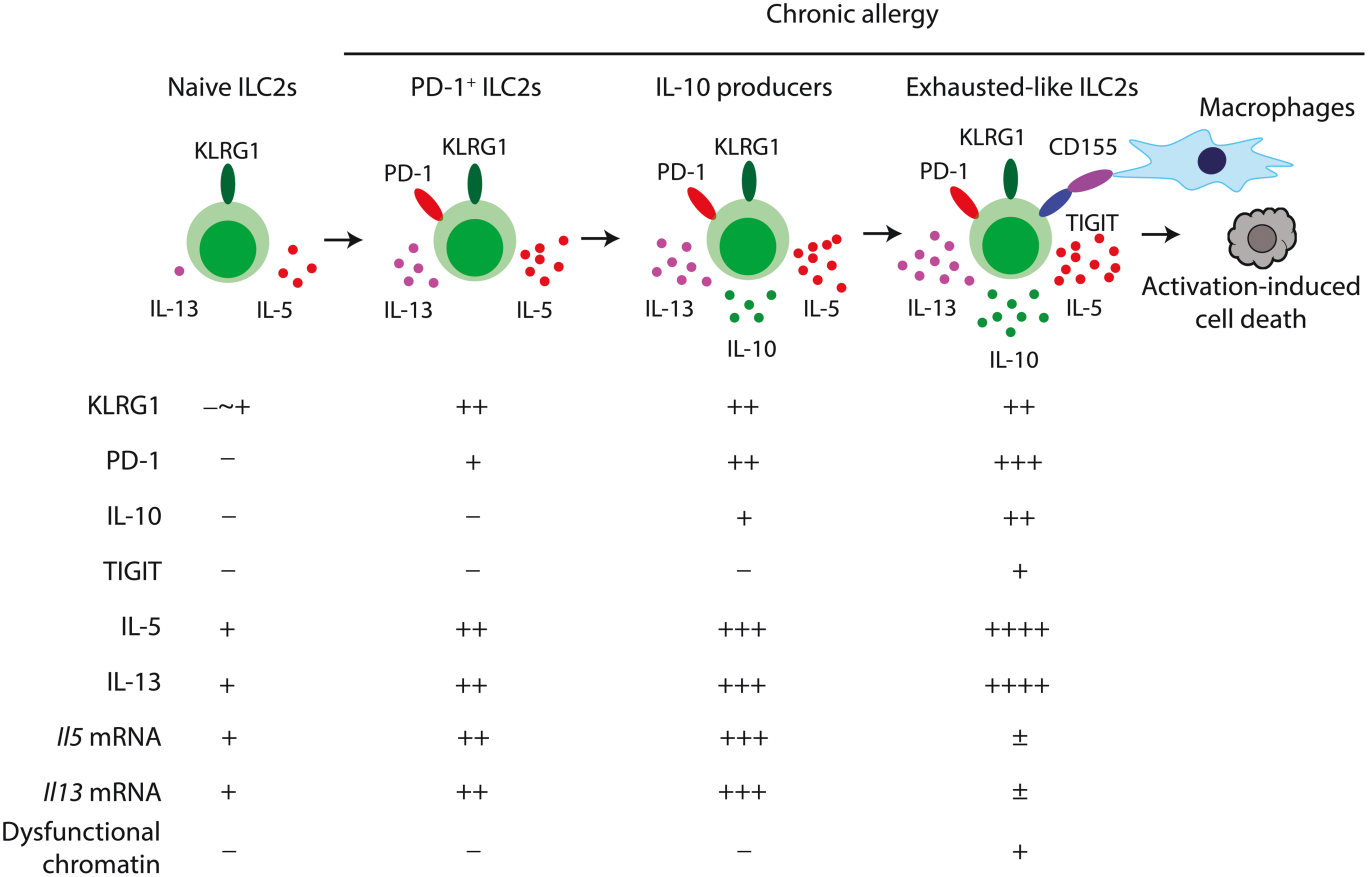
The ILC2 activation pathway to exhausted-like ILC2s and activation-induced cell death. Naïve ILC2s express a maturation/activation marker KLRG1. Though ILC2 progenitors in bone marrow are negative for KLRG1, around fifty percent of lung ILC2s and all intestinal ILC2s express KLRG1. PD-1 expression is quite low in naïve ILC2s but is induced in KLRG1^+^ ILC2s upon activation. Further ILC2 activation instigates IL-10 production in KLRG1^+^ PD-1^+^ ILC2s, followed by TIGIT expression and the emergence of exhausted-like ILC2s. Exhausted-like ILC2s quickly fall into activation-induced cell death via interaction with CD155-expressing alveolar macrophages.

Meanwhile, when the primarily activated Cbfβ-deficient ILC2s are stimulated with IL-33 *in vitro*, they exhibit decreased proliferation and cytokine production, suggesting that Cbfβ-deficient ILC2s are hyporesponsive to IL-33 (23). Transcriptional analysis demonstrates that the hyporesponsive ILC2s are associated with upregulation of *Tigit*, *Il10*, *Klrg1*, *Tnfrsf18* (GITR), *Prdm1* (Blimp-1), *Ctla4*, *Lag3*, and *Pdcd1*. Because of the expression of these markers and their dysfunction, KLRG1^+^ PD-1^+^ IL-10^+^ TIGIT^+^ ILC2s were tentatively named as exhausted-like ILC2s. The emergence of TIGIT^+^ IL-10^+^ exhausted-like ILC2s was precisely determined by Runx-competent IL-10-reporter mice. In the severe acute and chronic airway allergy model induced by papain nasal treatments, ILC2s in the bronchoalveolar fluid (BALF) were basically positive for KLRG1 and PD-1. Among the PD-1^+^ ILC2s, 30–40% of the cells produced IL-10-GFP (Fig. 4). TIGIT^+^ ILC2s were restricted to the PD-1^+^ IL-10^+^ population, consisting of a few percentages of all BALF ILC2s (23). The TIGIT^+^ ILC2s have low proliferative activity and low mRNA expression of *Il5* and *Il13*, indicating that TIGIT^+^ IL-10^+^ exhausted-like ILC2s can be induced by severe acute and chronic airway allergy even in mice with intact Runx protein function.

## AICD of ILC2s during chronic airway allergy

Because TIGIT^+^ ILC2s were not clearly segregated from TIGIT^−^ ILC2s by flow cytometry, TIGIT^+^ ILC2s were further analyzed using fate-tracer mice in which TIGIT-expressing cells can be marked by expression of tdTomato (a fluorescent protein) (21). In the model of chronic airway allergy induced by repeated nasal papain treatments, tdTomato^+^ TIGIT^+^ ILC2s were constantly generated as a 1–2% population of lung ILC2s. Phenotypical analysis suggested that the TIGIT^+^ ILC2s are more activated than TIGIT^−^ ILC2s because TIGIT^+^ ILC2s express more PD-1, IL-5–Venus, and IL-10–Venus (Venus is another fluorescent protein) than TIGIT^−^ ILC2s. However, transcriptome analysis indicated that global transcriptional arrest occurs in the coding region of TIGIT^+^ ILC2s. ILC2 signature genes including *Gata3*, *Il5*, and *Il13* were all dramatically downregulated and were overwhelmed by the expression of long non-coding RNA in the cells. In addition, downregulated genes were associated with reduced accessibility of the chromatin. Therefore, TIGIT^+^ ILC2s are highly activated cells having residual IL-5 and IL-10 but have shut-down functional mRNA expression because of dysfunctional chromatin changes.

Exhausted CD8 T cells accumulate in tumors or in tissues chronically infected with viruses (15). However, TIGIT^+^ ILC2s do not accumulate in tissues with chronic allergy; rather, the cells appear to be destined to die because highly active pan-caspases are detected in the TIGIT^+^ ILC2s (21). Adoptive transfer experiments demonstrated that TIGIT^+^ ILC2s are quickly removed from the airway within 1 hour. This may be the reason why only 1–2% of ILC2s are positive for TIGIT in the lung during chronic allergy despite continuous induction of the cells. Because TIGIT^+^ ILC2s are highly activated and are destined to die due to overwhelming activation, this type of cell death was tentatively called “ILC2 AICD.”

TIGIT on the exhausted-like ILC2s has a critical role in ILC2 AICD. Agonistic stimulation by anti-TIGIT antibody or CD155 on macrophages promotes cell death of TIGIT^+^ ILC2s *in vitro* (Fig. 4) (21). The importance of macrophages in ILC2 AICD was revealed by the facts that TIGIT^+^ ILC2s are co-localized with alveolar macrophages at the adventitial cuff of the lung and that deletion of alveolar macrophages increases the number of TIGIT^+^ ILC2s in the BALF during chronic airway allergy. Furthermore, the cell-autonomous functions of TIGIT in ILC2s have been elucidated by genetic ablation of *Tigit* in ILC2s. The absence of TIGIT increased the number of activated ILC2s but did not affect cytokine production, thereby deteriorating chronic allergy. Nevertheless, the cellular signaling mechanisms for ILC2 AICD remain elusive. TIGIT has immunoreceptor tyrosine-based inhibitory motif (ITIM) and immunoglobulin tail tyrosine (ITT) domains in the cytosol and inhibits the Akt–mTORC1 pathway (82). Because mTORC1 negatively regulates apoptosis, TIGIT signaling may be involved in cell death through mTORC1. Taken together, CD155^+^ macrophages constantly remove TIGIT^+^ exhausted-like ILC2s from the airway to decrease the number of activated ILC2s during chronic airway allergy.

## Conclusions

The success of immune checkpoint inhibitors in anti-tumor immunity pushes research to understand the CD8 T-cell exhaustion that dampens type 1 immune responses. The molecular basis of CD4 T-cell exhaustion in chronic LCMV infection has been also determined at a single-cell level. In contrast, the momentum for research to uncover exhaustion/dysfunction in type 2 immune responses is not strong probably because dysfunctional type 2 immune cells are not robust in chronic allergies. If dysfunctional Th2 cells dominate CD4 T cells in chronic allergy, the disease would be self-limiting. However, allergy tends to exacerbate, as shown in patients suffering from severe asthma and atopic dermatitis. Elucidating differences between type 1 and type 2 immune cell exhaustion/dysfunction may pave the way to the secret of the natural deteriorating trend in allergy.

It has not been clear whether exhaustion/dysfunction can be observed in type 3 immune cells, such as Th17 cells and ILC3 cells. Psoriasis is a good example of type 3 immune diseases caused by Th17 cells. However, single-cell analysis cannot demonstrate the presence of dysfunctional Th17 cells in psoriatic skin (83). Rather, TIM-3 expression is downregulated in peripheral blood Th17 cells from psoriasis patients, suggesting that exhausted Th17 cells may not be induced in psoriasis (84). PD-1^+^ ILC3s have been recently reported by Jacquelot *et al.* (85). PD-1 expression on ILC3s was induced by colitis caused by dextran sulfate sodium and suppressed IL-22 production in these cells. However, PD-1^+^ ILC3s may not be technically exhausted because they showed that PD-1^+^ ILC3s produced more IL-22 than PD-1^−^ ILC3s.

IL-10-producing ILC2s draw attention as a potential target for chronic allergy (69, 86). IL-33 and retinoic acid can generate IL-10-producing ILC2s among mouse and human ILC2s (69, 87, 88). Notably, antigen-specific immunotherapy induces IL-10-producing ILC2s in peripheral blood. The proportion of IL-10-producing ILC2s is negatively associated with clinical symptoms (86). However, IL-10 production can be a marker of ILC2 activation. In the murine model of airway allergy, IL-10-producing ILC2s produce high levels of IL-5 and IL-13 compared with non-IL-10 producers (69). More activation allows IL-10-producing ILC2s to acquire TIGIT and progressively produce IL-10 before AICD. Therefore, the immune-suppressive effect of IL-10-producing ILC2s may be in part mediated by AICD. Using ILC2-specific IL-10 deletion is necessary to clarify which is important for the regulatory function of IL-10-producing ILC2s: AICD or IL-10?

Exhausted-like TIGIT^+^ ILC2s are not technically exhausted because they do not accumulate in the allergic tissues. Instead of ILC2 exhaustion, hosts with chronic allergy need AICD of ILC2s to counteract excessive inflammation. There might be an alternative pathway for dysfunctional ILC2s as observed in CD8 T-cell exhaustion. However, the possibility might be unlikely because expression of inhibitory checkpoint receptors generally accumulates in the exhausted cells and such ILC2s expressing PD-1, TIGIT, and LAG-3 are exhausted-like ILC2s among activated ILC2s. It is not still clear whether the concept of TIGIT-mediated cell death can be applied to Th2 cells expressing TIGIT during chronic allergy. This hypothesis may be probable because dysfunctional Th2 cells express TIGIT and IL-10 and are phenotypically very similar to exhausted-like ILC2s. Promoting cell death in highly activated type 2 lymphocytes might be a good therapeutic avenue to mitigate chronic allergy.

## References

[1] Ebbo M , Crinier A, Vely F, et al. Innate lymphoid cells: major players in inflammatory diseases. Nat Rev Immunol 2017;17:665–78. 10.1038/nri.2017.86 28804130

[2] Vivier E , Artis D, Colonna M, et al. Innate lymphoid cells: 10 years on. Cell 2018;174:1054–66. 10.1016/j.cell.2018.07.017 30142344

[3] Martin-Fontecha A , Thomsen LL, Brett S, et al. Induced recruitment of NK cells to lymph nodes provides IFN-gamma for T(H)1 priming. Nat Immunol 2004;5:1260–5. 10.1038/ni1138 15531883

[4] Weizman OE , Adams NM, Schuster IS, et al. ILC1 confer early host protection at initial sites of viral infection. Cell 2017;171:795–808.e12. 10.1016/j.cell.2017.09.052 29056343 PMC5687850

[5] Kabata H , Moro K, Koyasu S. The group 2 innate lymphoid cell (ILC2) regulatory network and its underlying mechanisms. Immunol Rev 2018;286:37–52. 10.1111/imr.12706 30294963

[6] Christianson CA , Goplen NP, Zafar I, et al. 2015. Persistence of asthma requires multiple feedback circuits involving type 2 innate lymphoid cells and IL-33. J Allergy Clin Immunol 136:59. 25617223 10.1016/j.jaci.2014.11.037 PMC4494983

[7] Tojima I , Kouzaki H, Shimizu S, et al. Group 2 innate lymphoid cells are increased in nasal polyps in patients with eosinophilic chronic rhinosinusitis. Clin Immunol 2016;170:1–8. 10.1016/j.clim.2016.07.010 27422491

[8] Moro K , Yamada T, Tanabe M, et al. Innate production of T(H)2 cytokines by adipose tissue-associated c-Kit(+)Sca-1(+) lymphoid cells. Nature 2010;463:540–4. 10.1038/nature08636 20023630

[9] Neill DR , Wong SH, Bellosi A, et al. Nuocytes represent a new innate effector leukocyte that mediates type-2 immunity. Nature 2010;464:1367–70. 10.1038/nature08900 20200518 PMC2862165

[10] Halim TY , MacLaren A, Romanish MT, et al. Retinoic-acid-receptor-related orphan nuclear receptor alpha is required for natural helper cell development and allergic inflammation. Immunity 2012;37:463–74. 10.1016/j.immuni.2012.06.012 22981535

[11] Satoh-Takayama N , Vosshenrich CA, Lesjean-Pottier S, et al. Microbial flora drives interleukin 22 production in intestinal NKp46+ cells that provide innate mucosal immune defense. Immunity 2008;29:958–70. 10.1016/j.immuni.2008.11.001 19084435

[12] Sawa S , Cherrier M, Lochner M, et al. Lineage relationship analysis of RORgammat+ innate lymphoid cells. Science 2010;330:665–9. 10.1126/science.1194597 20929731

[13] Moskophidis D , Lechner F, Pircher H, et al. Virus persistence in acutely infected immunocompetent mice by exhaustion of antiviral cytotoxic effector T cells. Nature 1993;362:758–61. 10.1038/362758a0 8469287

[14] Zhang J , Lei F, Tan H. The development of CD8 T-cell exhaustion heterogeneity and the therapeutic potentials in cancer. Front Immunol 2023;14:1166128. 10.3389/fimmu.2023.1166128 37275913 PMC10232978

[15] Dolina JS , Van Braeckel-Budimir N, Thomas GD, et al. CD8(+) T cell exhaustion in cancer. Front Immunol 2021;12:715234. 10.3389/fimmu.2021.715234 34354714 PMC8330547

[16] Crawford A , Angelosanto JM, Kao C, et al. Molecular and transcriptional basis of CD4(+) T cell dysfunction during chronic infection. Immunity 2014;40:289–302. 10.1016/j.immuni.2014.01.005 24530057 PMC3990591

[17] Miggelbrink AM , Jackson JD, Lorrey SJ, et al. CD4 T-cell exhaustion: does it exist and what are its roles in cancer? Clin Cancer Res 2021;27:5742–52. 10.1158/1078-0432.CCR-21-0206 34127507 PMC8563372

[18] Taylor JJ , Krawczyk CM, Mohrs M, et al. Th2 cell hyporesponsiveness during chronic murine schistosomiasis is cell intrinsic and linked to GRAIL expression. J Clin Invest 2009;119:1019–28. 10.1172/JCI36534 19258704 PMC2662551

[19] van der Werf N , Redpath SA, Azuma M, et al. Th2 cell-intrinsic hypo-responsiveness determines susceptibility to helminth infection. PLoS Pathog 2013;9:e1003215. 10.1371/journal.ppat.1003215 23516361 PMC3597521

[20] Knipper JA , Ivens A, Taylor MD. Helminth-induced Th2 cell dysfunction is distinct from exhaustion and is maintained in the absence of antigen. PLoS NeglTrop Dis 2019;13:e0007908. 10.1371/journal.pntd.0007908 PMC6922449 31815932

[21] Yamada T , Tatematsu M, Takasuga S, et al. TIGIT mediates activation-induced cell death of ILC2s during chronic airway allergy. J Exp Med 2023;220:e20222005. 10.1084/jem.20222005 37036426 PMC10098142

[22] Ebihara T , Taniuchi I. Exhausted-like Group 2 innate lymphoid cells in chronic allergic inflammation. Trends Immunol 2019;40:1095–104. 10.1016/j.it.2019.10.007 31735510

[23] Miyamoto C , Kojo S, Yamashita M, et al. Runx/Cbfbeta complexes protect group 2 innate lymphoid cells from exhausted-like hyporesponsiveness during allergic airway inflammation. Nat Commun 2019;10:447. 10.1038/s41467-019-08365-0 30683858 PMC6347616

[24] Wherry EJ , Kurachi M. Molecular and cellular insights into T cell exhaustion. Nat Rev Immunol 2015;15:486–99. 10.1038/nri3862 26205583 PMC4889009

[25] Beltra JC , Manne S, Abdel-Hakeem MS, et al. Developmental relationships of four exhausted CD8(+) T cell subsets reveals underlying transcriptional and epigenetic landscape control mechanisms. Immunity 2020;52:825–41.e8. 10.1016/j.immuni.2020.04.014 32396847 PMC8360766

[26] Miller BC , Sen DR, Al Abosy R, et al. Subsets of exhausted CD8(+) T cells differentially mediate tumor control and respond to checkpoint blockade. Nat Immunol 2019;20:326–36. 10.1038/s41590-019-0312-6 30778252 PMC6673650

[27] Shin H , Blackburn SD, Intlekofer AM, et al. A role for the transcriptional repressor Blimp-1 in CD8(+) T cell exhaustion during chronic viral infection. Immunity 2009;31:309–20. 10.1016/j.immuni.2009.06.019 19664943 PMC2747257

[28] Shao P , Li F, Wang J, et al. Cutting edge: Tcf1 instructs T follicular helper cell differentiation by repressing Blimp1 in response to acute viral infection. J Immunol 2019;203:801–6. 10.4049/jimmunol.1900581 31300510 PMC6684471

[29] Wu T , Shin HM, Moseman EA, et al. TCF1 is required for the T follicular helper cell response to viral infection. Cell Rep 2015;12:2099–110. 10.1016/j.celrep.2015.08.049 26365183 PMC4591235

[30] Shan Q , Hu S, Chen X, et al. Ectopic Tcf1 expression instills a stem-like program in exhausted CD8(+) T cells to enhance viral and tumor immunity. Cell Mol Immunol 2021;18:1262–77. 10.1038/s41423-020-0436-5 32341523 PMC8093427

[31] Hudson WH , Gensheimer J, Hashimoto M, et al. Proliferating transitory T cells with an effector-like transcriptional signature emerge from PD-1(+) stem-like CD8(+) T cells during chronic infection. Immunity 2019;51:1043–58.e4. 10.1016/j.immuni.2019.11.002 31810882 PMC6920571

[32] Scott AC , Dundar F, Zumbo P, et al. TOX is a critical regulator of tumour-specific T cell differentiation. Nature 2019;571:270–4. 10.1038/s41586-019-1324-y 31207604 PMC7698992

[33] Khan O , Giles JR, McDonald S, et al. TOX transcriptionally and epigenetically programs CD8(+) T cell exhaustion. Nature 2019;571:211–8. 10.1038/s41586-019-1325-x 31207603 PMC6713202

[34] Sen DR , Kaminski J, Barnitz RA, et al. The epigenetic landscape of T cell exhaustion. Science 2016;354:1165–9. 10.1126/science.aae0491 27789799 PMC5497589

[35] Blake, MK, O’Connell P, Aldhamen, YA. Fundamentals to therapeutics: epigenetic modulation of CD8(+) T Cell exhaustion in the tumor microenvironment. Front Cell Dev Biol 2022;10:1082195. 36684449 10.3389/fcell.2022.1082195 PMC9846628

[36] Philip M , Fairchild L, Sun L, et al. Chromatin states define tumour-specific T cell dysfunction and reprogramming. Nature 2017;545:452–6. 10.1038/nature22367 28514453 PMC5693219

[37] Aubert RD , Kamphorst AO, Sarkar S, et al. Antigen-specific CD4 T-cell help rescues exhausted CD8 T cells during chronic viral infection. Proc Natl Acad Sci USA 2011;108:21182–7. 10.1073/pnas.1118450109 22160724 PMC3248546

[38] Morou A , Palmer BE, Kaufmann DE. Distinctive features of CD4+ T cell dysfunction in chronic viral infections. Curr Opin HIV AIDS 2014;9:446–51. 10.1097/COH.0000000000000094 25023623 PMC4231289

[39] Hwang S , Cobb DA, Bhadra R, et al. Blimp-1-mediated CD4 T cell exhaustion causes CD8 T cell dysfunction during chronic toxoplasmosis. J Exp Med 2016;213:1799–818. 10.1084/jem.20151995 27481131 PMC4995081

[40] Ciucci T , Vacchio MS, Gao Y, et al. The emergence and functional fitness of memory CD4(+) T cells require the transcription factor Thpok. Immunity 2019;50:91–105.e4. 10.1016/j.immuni.2018.12.019 30638736 PMC6503975

[41] Fahey LM , Wilson EB, Elsaesser H, et al. Viral persistence redirects CD4 T cell differentiation toward T follicular helper cells. J Exp Med 2011;208:987–99. 10.1084/jem.20101773 21536743 PMC3092345

[42] Zander R , Khatun A, Kasmani MY, et al. Delineating the transcriptional landscape and clonal diversity of virus-specific CD4(+) T cells during chronic viral infection. Elife 2022;11:e80079. 10.7554/eLife.80079 36255051 PMC9629829

[43] Bi J , Tian Z. NK Cell Exhaustion. Front Immunol 2017;8:760. 10.3389/fimmu.2017.00760 28702032 PMC5487399

[44] Zhang C , Wang XM, Li SR, et al. NKG2A is a NK cell exhaustion checkpoint for HCV persistence. Nat Commun 2019;10:1507. 10.1038/s41467-019-09212-y 30944315 PMC6447531

[45] Jost S , Altfeld M. Evasion from NK cell-mediated immune responses by HIV-1. Microbes Infect 2012;14:904–15. 10.1016/j.micinf.2012.05.001 22626930 PMC3432664

[46] Sun C , Sun H, Zhang C, et al. NK cell receptor imbalance and NK cell dysfunction in HBV infection and hepatocellular carcinoma. Cell Mol Immunol 2015;12:292–302. 10.1038/cmi.2014.91 25308752 PMC4654321

[47] Jia H , Yang H, Xiong H, et al. NK cell exhaustion in the tumor microenvironment. Front Immunol 2023;14:1303605. 10.3389/fimmu.2023.1303605 38022646 PMC10653587

[48] Concha-Benavente F , Kansy B, Moskovitz J, et al. PD-L1 mediates dysfunction in activated PD-1(+) NK cells in head and neck cancer patients. Cancer Immunol Res 2018;6:1548–60. 10.1158/2326-6066.CIR-18-0062 30282672 PMC6512340

[49] Liu Y , Cheng Y, Xu Y, et al. Increased expression of programmed cell death protein 1 on NK cells inhibits NK-cell-mediated anti-tumor function and indicates poor prognosis in digestive cancers. Oncogene 2017;36:6143–53. 10.1038/onc.2017.209 28692048 PMC5671935

[50] Oppenheim DE , Roberts SJ, Clarke SL, et al. Sustained localized expression of ligand for the activating NKG2D receptor impairs natural cytotoxicity in vivo and reduces tumor immunosurveillance. Nat Immunol 2005;6:928–37. 10.1038/ni1239 16116470

[51] Gill S , Vasey AE, De Souza A, et al. 2012. Rapid development of exhaustion and down-regulation of eomesodermin limit the antitumor activity of adoptively transferred murine natural killer cells. Blood 119:5758. 22544698 10.1182/blood-2012-03-415364 PMC3382935

[52] Ni J , Wang X, Stojanovic A, et al. Single-cell RNA sequencing of tumor-infiltrating NK cells reveals that inhibition of transcription factor HIF-1alpha unleashes NK cell activity. Immunity 2020;52:1075–87.e8. 10.1016/j.immuni.2020.05.001 32445619

[53] Judge SJ , Murphy WJ, Canter RJ. Characterizing the dysfunctional NK cell: assessing the clinical relevance of exhaustion, anergy, and senescence. Front Cell Infect Microbiol 2020;10:49. 10.3389/fcimb.2020.00049 32117816 PMC7031155

[54] Zhang Q , Bi J, Zheng X, et al. Blockade of the checkpoint receptor TIGIT prevents NK cell exhaustion and elicits potent anti-tumor immunity. Nat Immunol 2018;19:723–32. 10.1038/s41590-018-0132-0 29915296

[55] He Y , Peng H, Sun R, et al. Contribution of inhibitory receptor TIGIT to NK cell education. J Autoimmun 2017;81:1–12. 10.1016/j.jaut.2017.04.001 28438433

[56] da Silva IP , Gallois A, Jimenez-Baranda S, et al. Reversal of NK-cell exhaustion in advanced melanoma by Tim-3 blockade. Cancer Immunol Res 2014;2:410–22. 10.1158/2326-6066.CIR-13-0171 24795354 PMC4046278

[57] Ardolino M , Azimi CS, Iannello A, et al. Cytokine therapy reverses NK cell anergy in MHC-deficient tumors. J Clin Invest 2014;124:4781–94. 10.1172/JCI74337 25329698 PMC4347250

[58] Alvarez M , Simonetta F, Baker J, et al. Regulation of murine NK cell exhaustion through the activation of the DNA damage repair pathway. JCI Insight 2019;4:e127729. 10.1172/jci.insight.127729 PMC6675585 31211693

[59] Torcellan T , Friedrich C, Doucet-Ladeveze R, et al. Circulating NK cells establish tissue residency upon acute infection of skin and mediate accelerated effector responses to secondary infection. Immunity 2024;57:124–40.e7. 10.1016/j.immuni.2023.11.018 38157853 PMC10783803

[60] Su L , Lineberry N, Huh Y, et al. A novel E3 ubiquitin ligase substrate screen identifies Rho guanine dissociation inhibitor as a substrate of gene related to anergy in lymphocytes. J Immunol 2006;177:7559–66. 10.4049/jimmunol.177.11.7559 17114425

[61] Cao S , Liu J, Song L, et al. The protooncogene c-Maf is an essential transcription factor for IL-10 gene expression in macrophages. J Immunol 2005;174:3484–92. 10.4049/jimmunol.174.6.3484 15749884 PMC2955976

[62] Ebihara T , Tatematsu M, Fuchimukai A, et al. Trained innate lymphoid cells in allergic diseases. Allergol Int 2021;70:174–80. 10.1016/j.alit.2020.11.007 33328130

[63] Van Dyken SJ , Nussbaum JC, Lee J, et al. A tissue checkpoint regulates type 2 immunity. Nat Immunol 2016;17:1381–7. 10.1038/ni.3582 27749840 PMC5275767

[64] Moro K , Kabata H, Tanabe M, et al. Interferon and IL-27 antagonize the function of group 2 innate lymphoid cells and type 2 innate immune responses. Nat Immunol 2016;17:76–86. 10.1038/ni.3309 26595888

[65] Maazi H , Patel N, Sankaranarayanan I, et al. ICOS:ICOS-ligand interaction is required for type 2 innate lymphoid cell function, homeostasis, and induction of airway hyperreactivity. Immunity 2015;42:538–51. 10.1016/j.immuni.2015.02.007 25769613 PMC4366271

[66] Meylan F , Hawley ET, Barron L, et al. The TNF-family cytokine TL1A promotes allergic immunopathology through group 2 innate lymphoid cells. Mucosal Immunol 2014;7:958–68. 10.1038/mi.2013.114 24368564 PMC4165592

[67] Galle-Treger L , Sankaranarayanan I, Hurrell BP, et al. Costimulation of type-2 innate lymphoid cells by GITR promotes effector function and ameliorates type 2 diabetes. Nat Commun 2019;10:713. 10.1038/s41467-019-08449-x 30755607 PMC6372786

[68] Nagashima H , Okuyama Y, Fujita T, et al. GITR cosignal in ILC2s controls allergic lung inflammation. J Allergy Clin Immunol 2018;141:1939–43.e8. 10.1016/j.jaci.2018.01.028 29427641

[69] Seehus CR , Kadavallore A, Torre B, et al. Alternative activation generates IL-10 producing type 2 innate lymphoid cells. Nat Commun 2017;8:1900. 10.1038/s41467-017-02023-z 29196657 PMC5711851

[70] Moriyama S , Brestoff JR, Flamar AL, et al. beta2-adrenergic receptor-mediated negative regulation of group 2 innate lymphoid cell responses. Science 2018;359:1056–61. 10.1126/science.aan4829 29496881

[71] Nagashima H , Mahlakoiv T, Shih HY, et al. Neuropeptide CGRP limits Group 2 innate lymphoid cell responses and constrains Type 2 inflammation. Immunity 2019;51:682–95.e6. 10.1016/j.immuni.2019.06.009 31353223 PMC6801073

[72] Zhou W , Toki S, Zhang J, et al. Prostaglandin I2 signaling and inhibition of Group 2 innate lymphoid cell responses. Am J Respir Crit Care Med 2016;193:31–42. 10.1164/rccm.201410-1793OC 26378386 PMC4731613

[73] Cephus JY , Stier MT, Fuseini H, et al. Testosterone attenuates Group 2 innate lymphoid cell-mediated airway inflammation. Cell Rep 2017;21:2487–99. 10.1016/j.celrep.2017.10.110 29186686 PMC5731254

[74] Duerr CU , McCarthy CD, Mindt BC, et al. Type I interferon restricts Type 2 immunopathology through the regulation of Group 2 innate lymphoid cells. Nat Immunol 2016;17:65–75. 10.1038/ni.3308 26595887 PMC9135352

[75] Otaki N , Motomura Y, Terooatea T, et al. Activation of ILC2s through constitutive IFNgamma signaling reduction leads to spontaneous pulmonary fibrosis. Nat Commun 2023;14:8120. 10.1038/s41467-023-43336-6 38097562 PMC10721793

[76] Taylor S , Huang Y, Mallett G, et al. PD-1 regulates KLRG1(+) group 2 innate lymphoid cells. J Exp Med 2017;214:1663–78. 10.1084/jem.20161653 28490441 PMC5461001

[77] Helou DG , Shafiei-Jahani P, Lo R, et al. PD-1 pathway regulates ILC2 metabolism and PD-1 agonist treatment ameliorates airway hyperreactivity. Nat Commun 2020;11:3998. 10.1038/s41467-020-17813-1 32778730 PMC7417739

[78] Hoyler T , Klose CS, Souabni A, et al. The transcription factor GATA-3 controls cell fate and maintenance of type 2 innate lymphoid cells. Immunity 2012;37:634–48. 10.1016/j.immuni.2012.06.020 23063333 PMC3662874

[79] Ebihara T , Seo W, Taniuchi I. Roles of RUNX complexes in immune cell development. Adv Exp Med Biol 2017;962:395. 28299670 10.1007/978-981-10-3233-2_24

[80] Ebihara T , Taniuchi I. Transcription factors in the development and function of Group 2 innate lymphoid cells. Int J Mol Sci 2019;20:1377. 10.3390/ijms20061377 30893794 PMC6470746

[81] Ebihara T , Song C, Ryu SH, et al. Runx3 specifies lineage commitment of innate lymphoid cells. Nat Immunol 2015;16:1124–33. 10.1038/ni.3272 26414766 PMC4618046

[82] Sato K , Yamashita-Kanemaru Y, Abe F, et al. DNAM-1 regulates Foxp3 expression in regulatory T cells by interfering with TIGIT under inflammatory conditions. Proc Natl Acad Sci U S A 2021;118:e2021309118. 10.1073/pnas.2021309118 34011606 PMC8166105

[83] Ma F , Plazyo O, Billi AC, et al. Single cell and spatial sequencing define processes by which keratinocytes and fibroblasts amplify inflammatory responses in psoriasis. Nat Commun 2023;14:3455. 10.1038/s41467-023-39020-4 37308489 PMC10261041

[84] Kanai Y , Satoh T, Igawa K, et al. Impaired expression of Tim-3 on Th17 and Th1 cells in psoriasis. Acta Derm Venereol 2012;92:367–71. 10.2340/00015555-1285 22294262

[85] Jacquelot N , Xiong L, Cao WHJ, et al. PD-1 regulates ILC3-driven intestinal immunity and homeostasis. Mucosal Immunol 2024. 10.1016/j.mucimm.2024.03.002 38492744

[86] Golebski K , Layhadi JA, Sahiner U, et al. Induction of IL-10-producing type 2 innate lymphoid cells by allergen immunotherapy is associated with clinical response. Immunity 2021;54:291–307.e7. 10.1016/j.immuni.2020.12.013 33450188

[87] Bando JK , Gilfillan S, Di Luccia B, et al. ILC2s are the predominant source of intestinal ILC-derived IL-10. J Exp Med 2020;217:e20191520. 10.1084/jem.20191520 31699824 PMC7041711

[88] Morita H , Kubo T, Ruckert B, et al. Induction of human regulatory innate lymphoid cells from group 2 innate lymphoid cells by retinoic acid. J Allergy Clin Immunol 2019;143:2190–201.e9. 10.1016/j.jaci.2018.12.1018 30682454

